# Feasibility, safety, and comfort of the ‘2BB’ (2 cm below the bra) position of insertable cardiac monitors in women: the IN-WOMEN-ICM pilot study

**DOI:** 10.1093/europace/euae080

**Published:** 2024-04-03

**Authors:** F Javier Garcia-Fernández, Javier Martín González, Lola Villagraz Tercedor, Gonzalo Fernández Palacios, Daniel Cantero, Ermengol Vallés, Emilce Trucco

**Affiliations:** Arrythmia Unit, Cardiology Department, Hospital Universitario de Burgos, Avda Islas Baleares 3, 09006 Burgos, Spain; Arrythmia Unit, Cardiology Department, Hospital Universitario de Burgos, Avda Islas Baleares 3, 09006 Burgos, Spain; Arrythmia Unit, Cardiology Department, Hospital Universitario de Burgos, Avda Islas Baleares 3, 09006 Burgos, Spain; Arrythmia Unit, Cardiology Department, Hospital Universitario de Burgos, Avda Islas Baleares 3, 09006 Burgos, Spain; Arrythmia Unit, Cardiology Department, Hospital Universitario de Burgos, Avda Islas Baleares 3, 09006 Burgos, Spain; Arrythmia Unit, Cardiology Department, Hospital del Mar, Barcelona, Universidad Pompeu Fabra, Paseo marítimo de la Barceloneta 25, 08003 Barcelona, Spain; Arrythmia Unit, Cardiology Department, Hospital Universitario de Girona Dr Josep Trueta, Spain Avinguda de França, S/N, 17007 Girona, Spain; Institut d’Investigació Biomèdica de Girona (IDIBGI), Girona, Spain

**Keywords:** Insertable cardiac monitor, Insertion procedure, Inframammary, Women

## Abstract

**Graphical Abstract:**
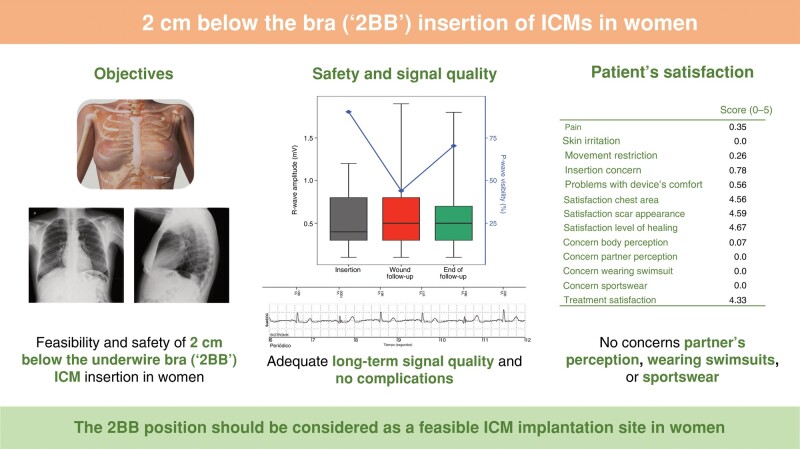
The left panel depicts a female thorax in supine position, highlighting the 2 cm below the underwire bra (2BB) inframammary insertion and chest X-ray images of a patient with the device inserted in the proposed position. The middle panel shows boxplots with median, Q1, Q3, Min, and Max values for the R-wave amplitude at insertion, wound follow-up, and end of follow-up (left axis) and the percentage of patients for whom P-waves were visible at standard resolution at insertion, wound follow-up, and end of follow-up (right axis), and an electrocardiogram recorded by a device implanted in the 2BB position. The right panel shows mean scores for each questionnaire item (0 = not at all, 5 = very much).

The left panel depicts a female thorax in supine position, highlighting the 2 cm below the underwire bra (2BB) inframammary insertion and chest X-ray images of a patient with the device inserted in the proposed position. The middle panel shows boxplots with median, Q1, Q3, Min, and Max values for the R-wave amplitude at insertion, wound follow-up, and end of follow-up (left axis) and the percentage of patients for whom P-waves were visible at standard resolution at insertion, wound follow-up, and end of follow-up (right axis), and an electrocardiogram recorded by a device implanted in the 2BB position. The right panel shows mean scores for each questionnaire item (0 = not at all, 5 = very much).

Insertable cardiac monitor (ICM) placement at standard anatomical positions (parasternal or parallel to the heart’s long axis) may lead to injury to the mammary gland, discomfort from purse or underwire bras, and aesthetic concerns because of the visible scar and bump.^1–3^ Moreover, the presence of an implanted medical device during mammography is linked to increased pain and anxiety, and suboptimal image quality.^4,5^

Subcutaneous inframammary insertion of ICMs could be an alternative position to avoid glandular issues and improve comfort and aesthetic appearance.^6–10^ However, only one study showed the feasibility of inserting ICMs above the natural breast fold area in the subcutaneous tissue in nine women, with adequate signal quality and no short-term complications.^2^

The ‘INWOMEN-ICM’ was a multicentre, prospective, single-arm, open-label pilot study to evaluate the feasibility of inserting ICMs 2 cm below the underwire bra (2BB). The study was conducted at three centres in Spain after approval from the Ethics Committee of participating centres and following the ethical principles of the Declaration of Helsinki. All participants provided written informed consent.

The device (BIOMONITOR III, Biotronik, Berlin, Germany) was inserted in a subcutaneous 2BB inframammary position by qualified physicians in the electrophysiology laboratory/operating room or ambulatory using local anaesthesia. The procedure was performed following the manufacturer’s instructions by employing the dedicated tools of the device with an insertion angulation between 30 and 45°.

Women aged >18 years with an ICM indication were included in the study. Patients were excluded when they had an indication for pacemaker or implantable cardioverter defibrillator, an axillary implant, a life expectancy < 12 months, participated in another interventional study, or were pregnant or breastfeeding.

The feasibility of performing 2BB inframammary ICM insertions in women was evaluated by assessing the insertion success rate, the incidence of procedure-related complications and wound-related adverse events, as well as long-term complications, comfort, and signal stability.

Data were collected at baseline, at patient’s discharge, at the wound follow-up visit (around 10 days after the insertion), and at the end of follow-up (9 months after the insertion). Device interrogations were performed at patient’s discharge, at the wound follow-up visit, and at the end of follow-up to retrieve the R-wave amplitude and P-wave visibility and arrhythmias detected. Patients rated their satisfaction level with the procedure via a purpose-made questionnaire comprising 13 items that could be scored from 0 (not at all) to 5 (very much). Since this was a pilot study, a formal sample size calculation was not required. However, including 30 patients was considered sufficient to assess the objectives of the study.^2,11,12^ Statistical analyses were performed using the SAS software (SAS Institute, Cary, SC, USA) for Windows, version 9.4.

Thirty-one women were included in the study [median age, 65 years; interquartile range (IQR), 44–74 years] and 29 completed the 9-month follow-up. All the insertions were successful, with no complications reported during the procedure. The median time from skin cut to insertion tool removal was 2 min (IQR, 1–4 min). Nineteen (61.3%) physicians considered the insertion procedure ‘very easy’, seven (22.6%) ‘easy’, and five (16.1%) ‘normal’ (*Table 1*).

**Table 1:** Insertion data

Variables	*n* = 31
ICM indication^a^, *n* (%)	
Syncope of unknown cause	14 (45.2%)
Cryptogenic stroke	7 (22.6%)
Suspected AF	4 (12.9%)
Risk factors	3 (9.7%)
Recurrent palpitations	3 (9.7%)
Ischaemic	2 (6.5%)
Atrioventricular conduction system disease	2 (6.5%)
Other	5 (16.1%)
Place of procedure, *n* (%)	
Electrophysiology laboratory/operating room	17 (54.8%)
Ambulatory	13 (41.9%)
Other	1 (3.2%)
Insertion time (skin cut to insertion tool removal, min), mean ± SD	3.0 ± 2.7
Median (IQR)	2.0 (1.0–4.0)
Wound closure methods, *n* (%)	
Staples	20 (64.5%)
Adhesive strips	6 (19.4%)
Intradermal stitches	5 (16.1%)
Ease of insertion, *n* (%)	
Very easy	19 (61.3%)
Easy	7 (22.6%)
Normal	5 (16.1%)
Difficult	0
Very difficult	0
Complications, *n* (%)	
None	31 (100%)
R-wave amplitude (mV), mean ± SD	0.56 ± 0.32
Median (IQR)	0.4 (0.3–0.8)
P-wave visibility, *n* (%)	28 (90.3%)

Data are expressed as mean ± SD, median (IQR), or *n* (%).

IQR, interquartile range; SD, standard deviation; AF, atrial fibrillation.

^a^Patients could present more than one cause/comorbidity.

No wound-related adverse event was reported 10 days after the insertion. The mean R-wave amplitude was 0.63 ± 0.47 mV (median, 0.50 mV; IQR, 0.30–0.80 mV), and P-waves were visible in 11 (44.0%) patients (*Graphical Abstract*).

At the end of follow-up, the mean R-wave amplitude was 0.58 ± 0.37 mV (median, 0.50 mV; IQR, 0.30–0.70 mV), and P-waves were visible in 19 (70.4%) patients (*Graphical Abstract*). The only wound-related adverse event was skin irritation. A total of 21 arrhythmias were detected. One patient (3.4%) was deemed a candidate for a pacemaker, while another (3.4%) for an implantable cardioverter defibrillator, and two cases (6.9%) required anticoagulation. No device was explanted. One patient underwent mammography during the follow-up, with no complications reported during the procedure.

The overall satisfaction with the procedure was high (score = 4.33). The items related to the appearance of the chest area (score = 4.56), the scar (score = 4.59), and the level of healing (score = 4.67) received the highest scores. Patients expressed no concerns regarding their partner’s perception, of wearing swimsuits or sportswear (*Graphical Abstract*).

In this pilot study, 2BB inframammary insertion of ICMs seemed a feasible option for women, with no complications, high acceptance, and good long-term signal quality. Inframammary ICM insertions were fast, requiring a median time of 2 min from incision to insertion tool removal. This time is in the range of that needed in a recent large cohort of patients with the same device inserted in standard anatomical positions (1 min from skin cut to tool removal)^13^ and lower than in a prospective observational study with another new generation ICM (7 min).^14^ The short procedural time reported in our study agrees with physicians’ positive rating of the insertion procedure, as previously observed.^15^ Acute and long-term device data revealed good signal quality and stability over time, with similar a R-wave amplitude^16^ and P-wave visibility^13,16^ at insertion as previously reported. Inframammary ICM insertions were safe, with no complications reported during the procedure. Similarly, other studies using the same device revealed a low rate of complications.^13,16^

Notably, women often experience more body image issues than men after implantable cardioverter defibrillator insertion, particularly related to the way that clothes fit over the device or wearing a bathing suit.^1^ In our study, patients reported no concerns regarding their partner’s perception, of wearing swimsuits, or sportswear. Additionally, they reported minimal to no perception of various symptoms associated with discomfort.

Taken together, the results of this pilot study are comparable to those reported with devices at standard positions, but inframammary insertions may offer women no risk of complications in the mammary gland, greater comfort, better cosmetic outcomes, and eventually reduce pain and anxiety during mammography. The main limitations of the study are the small sample size, the lack of comparator, and that results are restricted to a specific device from a single manufacturer.

## Conclusion

2BB inframammary insertions of ICMs were fast, showed adequate long-term signal quality, no complications, and high patient acceptance and comfort. When performing an ICM insertion for women, it is crucial to consider the 2BB position as a feasible option.

## Data Availability

Data are available upon reasonable request.
